# Follow-Up Care for Breast and Colorectal Cancer Across the Globe: Survey Findings From 27 Countries

**DOI:** 10.1200/GO.20.00180

**Published:** 2020-09-21

**Authors:** Michelle A. Mollica, Deborah K. Mayer, Kevin C. Oeffinger, Youngmee Kim, Susan S. Buckenmaier, Sudha Sivaram, Catherine Muha, Nur Aishah Taib, Elisabeth Andritsch, Chioma C. Asuzu, Ovidiu V. Bochis, Sheila Diaz, Maria Die Trill, Patricia J. Garcia, Luigi Grassi, Yosuke Uchitomi, Asim Jamal Shaikh, Michael Jefford, Hyun Jeong Lee, Christoffer Johansen, Emmanuel Luyirika, Elizabeth Jane Maher, Maria Madeline B. Mallillin, Theoneste Maniragaba, Anja Mehnert-Theuerkauf, C. S. Pramesh, Sabine Siesling, Orit Spira, Jonathan Sussman, Lili Tang, Nguyen V. Hai, Suayib Yalcin, Paul B. Jacobsen

**Affiliations:** ^1^ National Cancer Institute Bethesda, MD; ^2^ University of North Carolina at Chapel Hill, Chapel Hill, NC; ^3^ Duke University School of Medicine, Durham, NC; ^4^ University of Miami, Coral Gables, FL; ^5^ Graddip Genet Counsell, University of Malaya, Kuala Lumpur, Malaysia; ^6^ Medizinische Universitat Graz, Graz, Austria; ^7^ University of Ibadan, Ibadan, Nigeria; ^8^ Universitatea de Medicina si Farmacie Iuliu Hatieganu Facultatea de Medicina, Cluj-Napoca, Romania; ^9^ Universidad Autonoma de Santo Domingo Facultad Ciencias de la Salud, Santo Domingo, Dominican Republic; ^10^ Hospital General Universitario Gregorio Maranon, Madrid, Spain; ^11^ Universidad Peruana Cayetano Heredia, Lima, Peru; ^12^ University of Ferrara, Ferrara, Italy; ^13^ National Cancer Center Hospital, Tokyo, Japan; ^14^ Aga Khan University, Karachi, Pakistan; ^15^ Peter MacCallum Cancer Centre, Melbourne, Victoria, Australia; ^16^ National Cancer Center, Goyang, Korea; ^17^ Rigshospitalet, University of Copenhagen, Copenhagen, Denmark; ^18^ African Palliative Care Association, Kampala, Uganda; ^19^ Macmillan Cancer Support, London, United Kingdom; ^20^ Asian Hospital and Medical Center, Muntinlupa, Philippines; ^21^ Republic of Rwanda Ministry of Health, Kigali, Rwanda; ^22^ Universitat Medical Center Leipzig, Leipzig, Germany; ^23^ Tata Memorial Centre, Mumbai, India; ^24^ Netherlands Comprehensive Cancer Organisation and University of Twente, Enschede, the Netherlands; ^25^ Israel Cancer Association, Givatayim, Israel; ^26^ McMaster University, Hamilton, Ontario, Canada; ^27^ Peking University Cancer Hospital, Beijing, People’s Republic of China; ^28^ Ho Chi Minh City University of Medicine and Pharmacy, Ho Chi Minh City, Vietnam; ^29^ Hacettepe University Institute of Cancer, Ankara, Turkey

## Abstract

**PURPOSE:**

The purpose of this study was to describe follow-up care for breast and colorectal cancer survivors in countries with varying levels of resources and highlight challenges regarding posttreatment survivorship care.

**METHODS:**

We surveyed one key stakeholder from each of 27 countries with expertise in survivorship care on questions including the components/structure of follow-up care, delivery of treatment summaries and survivorship care plans, and involvement of primary care in survivorship. Descriptive analyses were performed to characterize results across countries and variations between the WHO income categories (low, middle, high). We also performed a qualitative content analysis of narratives related to survivorship care challenges to identify major themes.

**RESULTS:**

Seven low- or /lower-middle-income countries (LIC/LMIC), seven upper-middle-income countries (UMIC), and 13 high-income countries (HICs) were included in this study. Results indicate that 44.4% of countries with a National Cancer Control Plan currently address survivorship care. Additional findings indicate that HICs use guidelines more often than those in LICs/LMICs and UMICs. There was great variation among countries regardless of income level. Common challenges include issues with workforce, communication and care coordination, distance/transportation issues, psychosocial support, and lack of focus on follow-up care.

**CONCLUSION:**

This information can guide researchers, providers, and policy makers in efforts to improve the quality of survivorship care on a national and global basis. As the number of cancer survivors increases globally, countries will need to prioritize their long-term needs. Future efforts should focus on efforts to bridge oncology and primary care, building international partnerships, and implementation of guidelines.

## INTRODUCTION

The number of people living with and beyond a cancer diagnosis has risen exponentially over the recent years and is expected to continue to grow. In 2018, it was estimated that 18.1 million new cases of cancer were diagnosed throughout the world,^1^ with this number expected to grow to 24 million by 2035.^2^ Moreover, less-developed countries have recently experienced large increases in the number of survivors as systematic efforts in screening and detection contribute to earlier diagnosis and navigation to treatment.

**CONTEXT**

**Key Objective**
How is survivorship care for breast and colorectal cancer survivors delivered globally among low-, middle-, and high-income countries?
**Knowledge Generated**
Results indicated great variation among countries regardless of income levels. Stakeholders reported challenges with workforce capacity, communication and care coordination, and lack of focus on follow-up care.
**Relevance**
Attention to monitoring for recurrence, subsequent malignancies, and persistent issues for cancer survivors is essential to maximize the benefits of curative and life-extending treatments.

Although advances in diagnostic and treatment capabilities have improved cancer survival, the issues facing people after completion of initial cancer treatment have only more recently been recognized. Evidence demonstrates that many cancer survivors are at increased risk for late and long-term effects well after the end of treatment, including cardiovascular disease, second cancers, fatigue, and other physiologic and psychosocial sequelae that impact quality of life,^3^ ability to return to work,^4^ and increased costs to the health care system.^5^ Targeted interventions that address survivor needs have been shown through numerous trials to improve many of these issues.^6,7^ Overall, provision of survivorship care has the potential to improve quality of life and functioning in individual patients, result in improved health care utilization, and potentially reduce morbidity and improve survival through surveillance for recurrence and identifying and addressing persistent issues.

The US Institute of Medicine (IOM), now the National Academy of Medicine, identified the following as important components of posttreatment survivorship care:

1. Prevention and surveillance for recurrence and subsequent primary cancers
2. Surveillance and management of physical and psychosocial effects of cancer and its treatment
3. Health promotion and preventive care
4. Care coordination among providers and with survivors.^8^

Although there have been increasing efforts to enhance survivorship care through international discussions and collaborations,^9,10^ there is a lack of information on the delivery of posttreatment follow-up care for survivors across a wide range of countries. Previous research has focused on high-income countries or is limited to one region.^11^ A recent review on the state of survivorship care for childhood-onset cancers across 18 countries highlighted challenges in communication and coordination, particularly in the transition between pediatric and adult care and among low-resource settings.^12^

By reviewing current practices and challenges in delivering appropriate follow-up care for survivors of adult-onset cancers, particularly across low-, middle-, and high-income countries, we can begin to understand commonalities and variations. We recognize that under-resourced countries may have competing priorities in the advancement of cancer-related care. However, as under-resourced countries increasingly adopt resource-stratified guidelines for the treatment of cancer, there is a societal and ethical obligation to initiate monitoring for recurrence and subsequent malignancies and management of long-term and late effects in those who received treatment. Accordingly, collecting data about the status of survivorship at the national level and addressing survivorship care as part of a national cancer control plan can help health care providers, researchers, and policy makers in low-resource settings determine where initial efforts in this area should be directed. For countries with greater resources, this information can serve as foundational for furthering the development of international survivorship networks and dissemination and adoption of evidence-based guidelines for survivorship care.

With these goals in mind, the purpose of this study was to: (1) describe models of survivorship care from countries with varying income levels, and (2) highlight challenges to delivering posttreatment follow-up care for breast and colorectal cancer survivors. Because it is likely that there is great heterogeneity of survivorship care among cancer sites, this survey targeted breast and colorectal cancers, as these cancers are highly prevalent and guidelines for follow-up care are established and available.^13,14^

## METHODS

### Identification of Countries and Stakeholders

To achieve a descriptive global survey, we used a convenience sample method with an effort to include multiple countries across different continents and with adequate representation of low- and middle-income countries. One stakeholder per country was selected based on their broad understanding of survivorship care in their country and their role in leading survivorship research and/or clinical efforts at a government or institutional level. Of 28 national stakeholders approached, 27 agreed to participate (96%). Stakeholders were invited to complete a survey and asked to provide a brief narrative on the challenges in delivering posttreatment follow-up care in their country (survey can be found in the Appendix). Stakeholders were asked to make concerted efforts to determine that responses reflect the pattern of care across the country.

All procedures performed in studies involving human participants were in accordance with the ethical standards of the institutional and/or national research committee and with the 1964 Helsinki declaration and its later amendments or comparable ethical standards.

Although we acknowledge different definitions of “cancer survivor,” we asked stakeholders to reflect on care for those who have completed active treatment with curative intent and are in the posttreatment period. Stakeholders were also asked to respond based on common practices for their country, recognizing that there may be variability within their country. The current survey was adapted from a survey on models of care of childhood cancer survivors,^12^ pilot tested with a subset of four countries, and then delivered electronically to all stakeholders.

### Analysis

We categorized countries into the following groups: low/lower-middle income (LIC/LMIC), upper-middle income (UMIC), and high income (HIC), based on the World Bank classification of economies to identify the income category for each country.^15^ We performed a descriptive analysis of quantitative survey items to describe frequencies and patterns by income category. We analyzed the open-ended question on challenges to delivering survivorship care using a directed content analysis approach.^16^ Two coauthors (M.A.M., S.S.B.) coded each response by labeling or marking text segments with the appropriate theme. The text segments were then reread, and more fine-grained codes were developed.^6^ This process was repeated until all the responses were assigned fine-grained codes. Discrepancies in assignment of codes were resolved through discussion and consensus.

## RESULTS

### Overview

**FIG 1:**
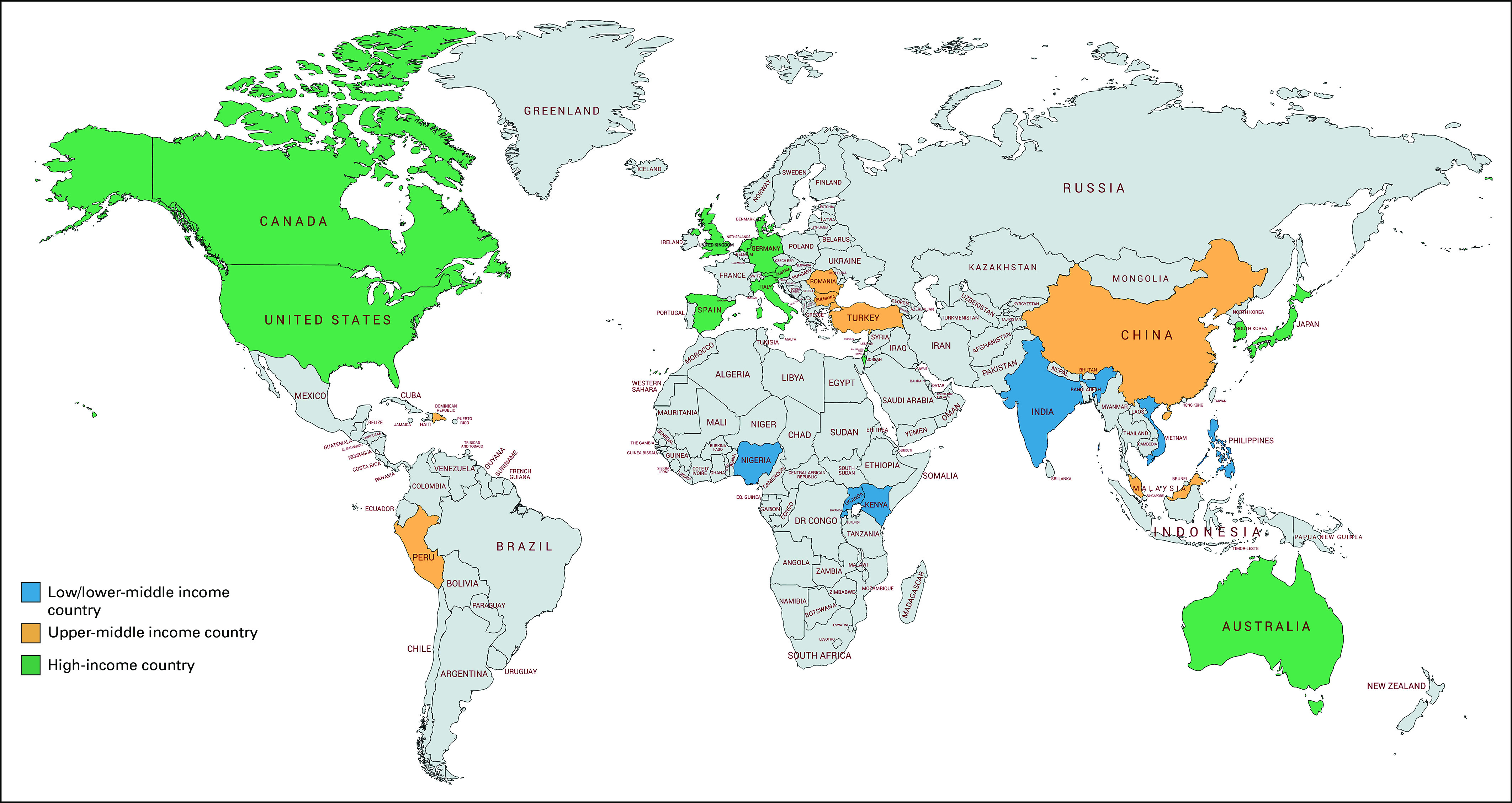
Countries included in global follow-up care for breast and colorectal cancer survivors survey (N = 27).

The final sample included 27 countries from six continents and consisted of seven LICs/LMICs, seven UMICs, and 13 HICs (Fig 1). The stakeholders included clinicians (67.9%), researcher/scientists (17.9%), and clinician researchers (14.3%) with specific expertise in breast and colorectal cancer survivorship care. Clinical disciplines included oncologists, psychologists, social workers, and nurse practitioners.

Survey results varied greatly among countries, and, as such, we report aggregate results in Tables 1-4 and individual country data in Appendix Tables A1-A4. Below, we highlight key findings across countries, as well as notable differences by income category. Among the 22 countries that have National/Governmental Cancer Control Plans (NCCPs), survivorship care is included in 12 countries (54.6%), is under development in five countries (22.7%), and is not in development in five countries (22.7%).

**TABLE 1:**
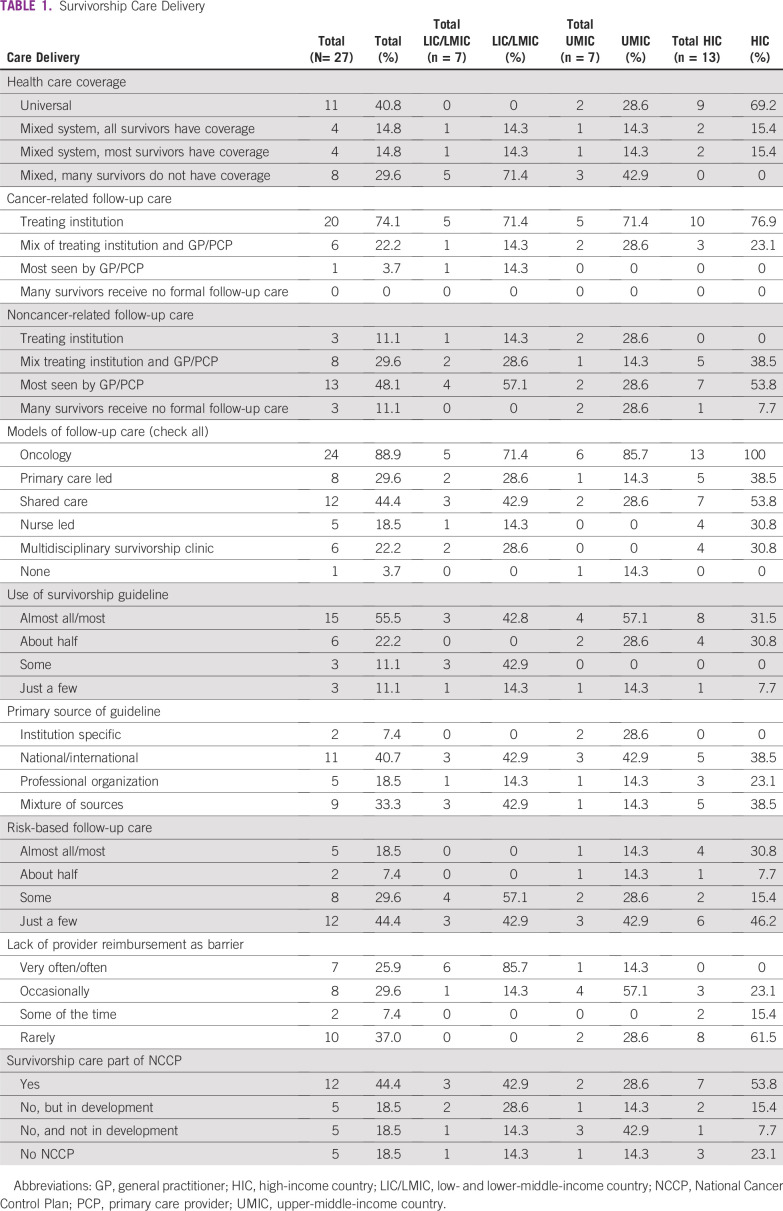
Survivorship Care Delivery

**TABLE 2:**
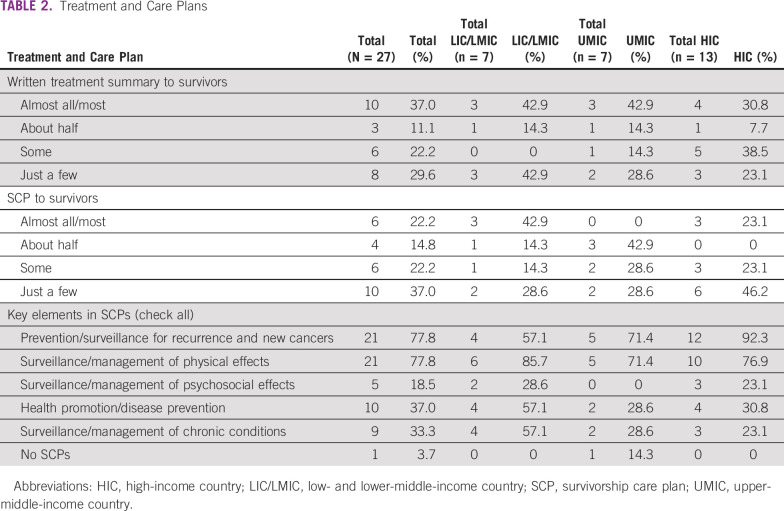
Treatment and Care Plans

**TABLE 3:**
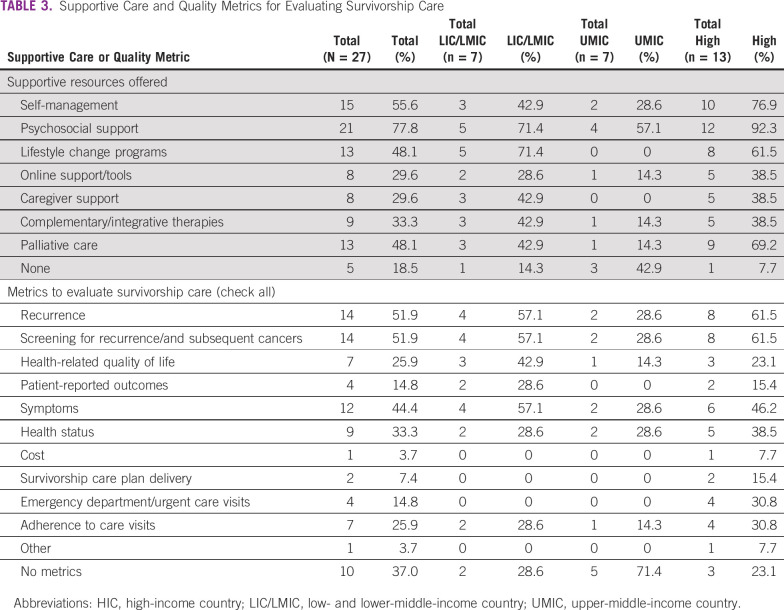
Supportive Care and Quality Metrics for Evaluating Survivorship Care

**TABLE 4:**
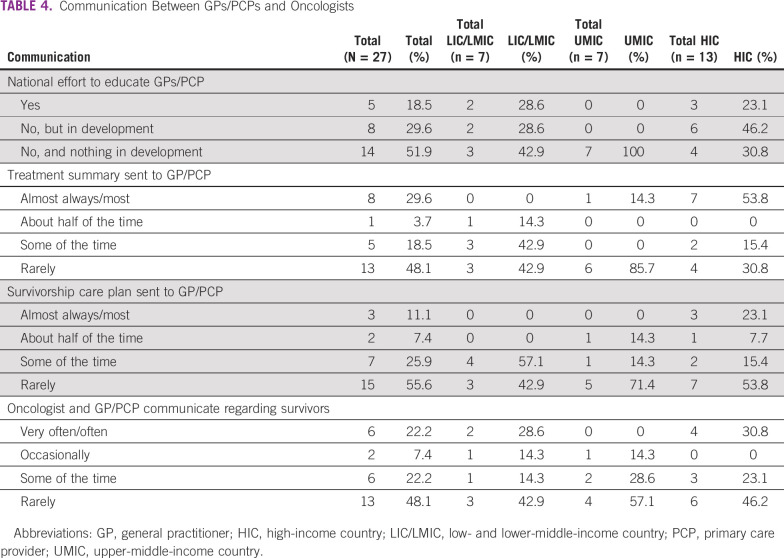
Communication Between GPs/PCPs and Oncologists

### Survivorship Care Delivery and Structure

Across countries, 24 (88.9%) use oncology-led follow up care, and 12 (44%) use shared-care models with the oncology team and general practitioner/primary care provider (GP/PCP; Table 1). Cancer-related follow-up care is most often delivered through the treating institution (74.1%; n = 20), whereas noncancer-related follow-up care is most often delivered by a GP/PCP (48.1%; n = 13). We found these patterns to be generally true for cancer-related care in LICs/LMICs, UMICs, and HICs and for noncancer LICs/LMICs and HICs. In contrast, the site where noncancer-related follow-up care is delivered in UMICs is more variable, where noncancer follow-up care is delivered mainly by the treating institution in two countries (28.6%), by the GP/PCP in two countries (28.6%), or with no formal follow-up care in two countries (28.6%).

In 15 countries (55.6%), most clinicians use guidelines to inform posttreatment follow-up care; the use of guidelines varied by income level. Guideline were used in eight (61.5%) of HICs, four (57.1%) of UMICs, and three (42.8%) of LICs/LMICs. An additional item asked about risk-stratified survivorship care that considers patients’ needs and their complexity when deciding which survivors are seen at the cancer center versus in the community. This approach to care is used most of the time in only five (18.5%) of countries, with four of these countries being HICs. Finally, lack of reimbursement/compensation for follow-up care was very often or often a barrier in seven (25.9%) of countries; this was more often the case in LICs/LMICs (n = 6; 85.7%) than UMICs (n = 1; 14.3%) and HICs (n = 0).

### Treatment Summaries and Survivorship Care Plans

In 10 countries (37%), most survivors receive a written summary of their cancer treatment, and in six countries (22.2%), most survivors receive a written plan for posttreatment follow-up care (ie, survivorship care plan [SCP]; Table 2). We found a consistent pattern across income categories for distribution of treatment summaries. In contrast, the distribution of SCPs was more variable. In LICs/LMICs, three (42.9%) of countries, and in HICs, three (29.7%), provide care plans to most survivors. No UMICs report providing SCPs to most survivors. For those that provided SCPs, key elements of posttreatment follow-up care most often included prevention and surveillance for recurrence and new cancers (n = 21; 77.8%) and surveillance and management of physical effects of cancer and its treatment (n = 21; 77.8%).

### Supportive Programs and Quality Metrics for Survivors

As shown in Table 3, the most frequent supportive resources offered across countries are psychosocial support (n = 21; 77.8%), self-management resources (n = 15; 55.6%), lifestyle change programs (n = 13; 48.1%), and palliative care (n = 13; 48.1%). Five countries ( 18.5%) do not offer any support programs or resources to survivors after the completion of treatment. There are 10 countries (37%) that currently do not use any metrics to evaluate the quality of posttreatment follow-up care, including two LICs/LMICs, five UMICs, and three HICs. Of those countries that do use metrics, recurrence (n = 14; 51.9%), screening for recurrence and subsequent primary cancers (n = 14; 51.9%), and survivor symptoms (n = 12; 44.4%) were most often used.

### Involvement of Nononcology Providers and Communication Among Providers and Caregivers

Six countries (22.2%) have a national effort to educate GPs/PCPs about the needs and care of cancer survivors (Table 4). Of the remaining 21 countries, eight (38.1%) have a plan in development and 13 (61.9%) do not have a plan in development. Oncology teams and GPs/PCPs communicate to share information about survivors often or very often in six countries (22.2%). In eight countries (29.6%), the treating institution sends a copy of the cancer treatment summary to the GP/PCP most of the time or almost always; this finding is mostly driven by HICs, which comprise seven of the eight countries.

### Challenges in the Delivery of Follow-Up Care

Several themes emerged from the qualitative analysis of stakeholder narratives of challenges in posttreatment follow-up care (Table 5). These include:

1. Workforce issues
2. Communication and care coordination
3. Access issues
4. Psychosocial support issues
5. Lack of focus on follow-up care.

**TABLE 5:**
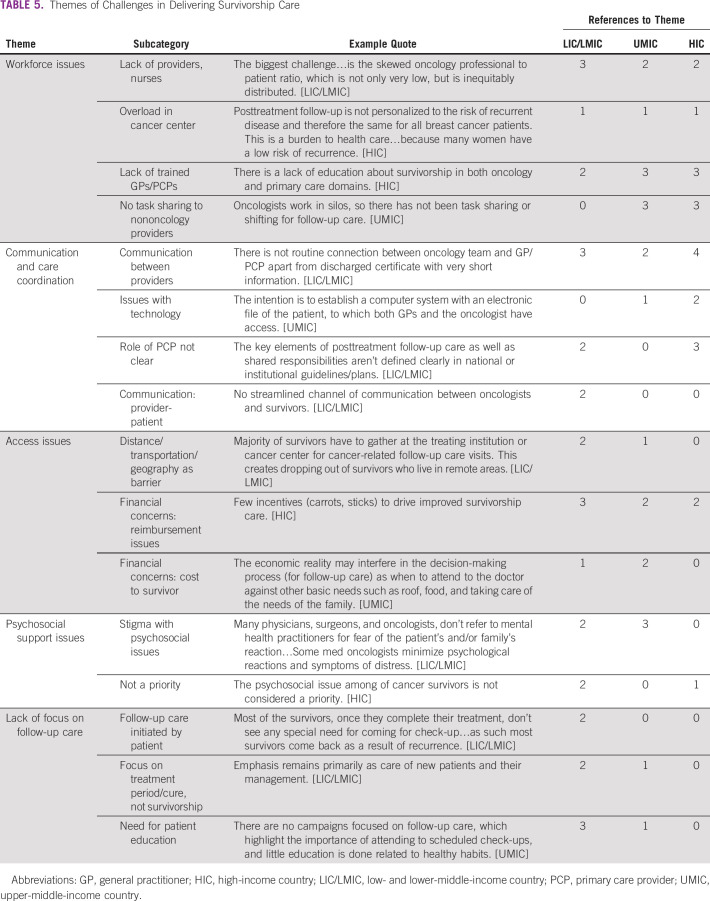
Themes of Challenges in Delivering Survivorship Care

Workforce issues included a shortage of providers, overload in cancer centers, a lack of trained PCPs, and issues with oncologists not sharing care with nononcology providers. Communication and care coordination issues included breakdowns in connections between providers, no streamlined communication between oncologists and survivors, issues with technology, and lack of role delineation with respect to the PCP. Access issues included distance to facility and transportation challenges, reimbursement issues for providers who deliver survivorship care, and financial costs to the survivor. Psychosocial support issues are also a challenge, with stigma surrounding psychosocial issues and lack of prioritization for psychosocial care central to this theme. Finally, there was a general lack of focus on follow-up care referenced. Two countries noted that follow-up care is initiated by the patient and they do not always see the need for continued care, and others indicated that there was a general focus on the treatment period and not on survivorship.

## DISCUSSION

This study sought to examine the delivery of posttreatment follow-up care for breast and colorectal cancer survivors across the globe. Our results extend previous work focused on single regions^11^ and high-income countries,^17^ which highlighted barriers to advancing survivorship care. Of note, we found that only 44.4% of 22 countries with an NCCP currently address survivorship care. This is slightly higher than a 2018 global analysis of NCCPs, which found that 32% of countries addressed survivorship in their NCCP.^18^ For those that do not have an NCCP, development of a plan with a survivorship component may be a first step in improving the quality of follow-up care.^19^ Below we offer additional recommendations on the basis of our study results.

Countries use several different models for the delivery of posttreatment follow-up care, including oncology-led, PCP-led, nurse-led, and shared care approaches as well as multidisciplinary survivorship clinics. In the absence of clear evidence supporting one model of survivorship care over others,^20^ the model delivered is often based on provider preferences, resources, and logistics. Although several publications identify potential advantages of risk-stratified^21^ and risk-based care,^22^ our results indicate that this approach is used in only one-fifth of the countries surveyed. One possible reason for this is the lack of consensus for features included in risk stratification and lack of readily available prediction models for key outcomes, although efforts to predict risk for recurrence are underway.^23^ We recommend that countries begin by directing resources toward surveillance for recurrence and second cancers for those at highest risk.

Our results also indicated that in slightly more than half of the countries, most clinicians use guidelines to inform posttreatment follow-up care for breast and colorectal cancer survivors. There was, however, less use of guidelines in LICs compared with UMICs and HICs. This situation may reflect the fact that most guidelines are developed in HICs and are designed for clinical settings that are often well-resourced and do not explicitly consider the needs of settings with fewer resources. Recognizing this situation, a number of organizations have developed resource-stratified guidelines that specify in an incremental manner different levels of care for surveillance for recurrence, second cancers, and persistent issues on the basis of available resources. The Breast Health Global Initiative has led one such effort, producing resource-stratified guidelines on breast health and cancer control, including survivorship and supportive care.^24^ Resource-stratified guidelines in colorectal cancer, to date, have focused on only early detection and treatment. Because the use of guidelines may enhance the quality of survivorship care and patient outcomes, our findings signal a need to consider resource-stratified survivorship guidelines for other cancer types.

The results of our survey showed that communication and coordination between oncology and nononcology providers and with patients and their families is a challenge for countries regardless of income category, with few countries reporting that oncologists and GPs/PCPs routinely communicate to share information. Our content analysis supported this finding, with stakeholders citing several issues with communication, including a lack of role clarity, no connection between the oncology team and GP/PCP, and issues with technology. Although the use of an SCP was recommended in the seminal IOM “Lost in Transition” report,^8^ our results showed that in only six countries do survivors routinely receive a written care plan for posttreatment follow-up care. Previous research indicates that implementation and evidence supporting SCPs has been mixed^25,26^; however, the SCP may be a starting point to enhancing coordination among providers. In addition, there have been efforts by organizations such as the Cancer and Research Primary Care International Network and ASCO to bring together oncology and nononcology providers through research and collaborative meetings. We recommend continued efforts to bridge the persistent gap between oncology and primary care.

Another key strategy in improving survivorship care globally is to systematically evaluate the quality of the care delivered. Our results indicate that 37% of countries do not use any metrics to evaluate survivorship care, and among those that do, almost all include recurrence and screening for recurrence as a key metric. Although these are important metrics, survivorship care should be holistic and often involves a multilevel evaluation of the comprehensive components of care. A recent evidence-based framework provides a starting point for future efforts to measure and improve cancer survivorship care.^27^

It is also important to note that many stakeholders emphasized the role of nongovernmental organization (NGO) support in survivorship and supportive care. Rehabilitation, psychological support, and other efforts in these countries may be driven by support from these organizations rather than governmental or federal organizations. Future research should include a global scan of the work of NGOs in supportive survivorship care across countries from varied income levels and health care systems.

This study provides critical data about posttreatment follow-up care for cancer survivors, including broad representation of LICs/LMICs and UMICs. The results should be viewed in light of certain caveats. First, findings are based on the responses of a single stakeholder from each country, each with different professional roles that may have affected responses. Although the stakeholder was identified as knowledgeable about survivorship efforts and encouraged to consult their peers, the extent to which these responses correspond to objective data on survivorship care, assuming it could be collected, remains unknown. Limiting each country to a single respondent also may obscure what may be considerable within-country variation in survivorship care. Regardless, this was an initial attempt to use key stakeholders to characterize survivorship care globally. In addition, although they have large and growing cancer survivor populations, South America and Africa were relatively under-represented continents in this study. Our team did make efforts to identify stakeholders with adequate expertise in other countries in these regions, however. Future work should continue to expand the evaluation of survivorship care in other countries globally. Finally, the number of countries surveyed was adequate to conduct descriptive analyses, but we were not able to conduct inferential statistical analyses based on income category. Recognizing these limitations, future studies should focus on identifying multiple stakeholders from each country to better represent the variation within and among countries and regions.

In summary, our findings identify several opportunities for enhancing survivorship care on a global basis, including the use of resource-stratified guidelines as well as the inclusion of survivorship care in NCCPs. In addition, there is a need to increase efforts to improve communication and coordination between oncology and nononcology providers. We recognize that each country may have different priorities for cancer control and that efforts to improve screening and/or treatment outcomes may be more pressing than a focus on survivorship care in the context of limited health resources. As countries invest in curative and life-extending cancer treatments, however, attention to monitoring for recurrence and subsequent malignancies is crucial if the benefits of these investments are to be fully realized. In addition to helping preserve treatment gains, the delivery of quality follow-up care has the potential to address issues that may affect survivors’ ability to return to work or resume important social roles.

## Figures and Tables

**TABLE A1:**
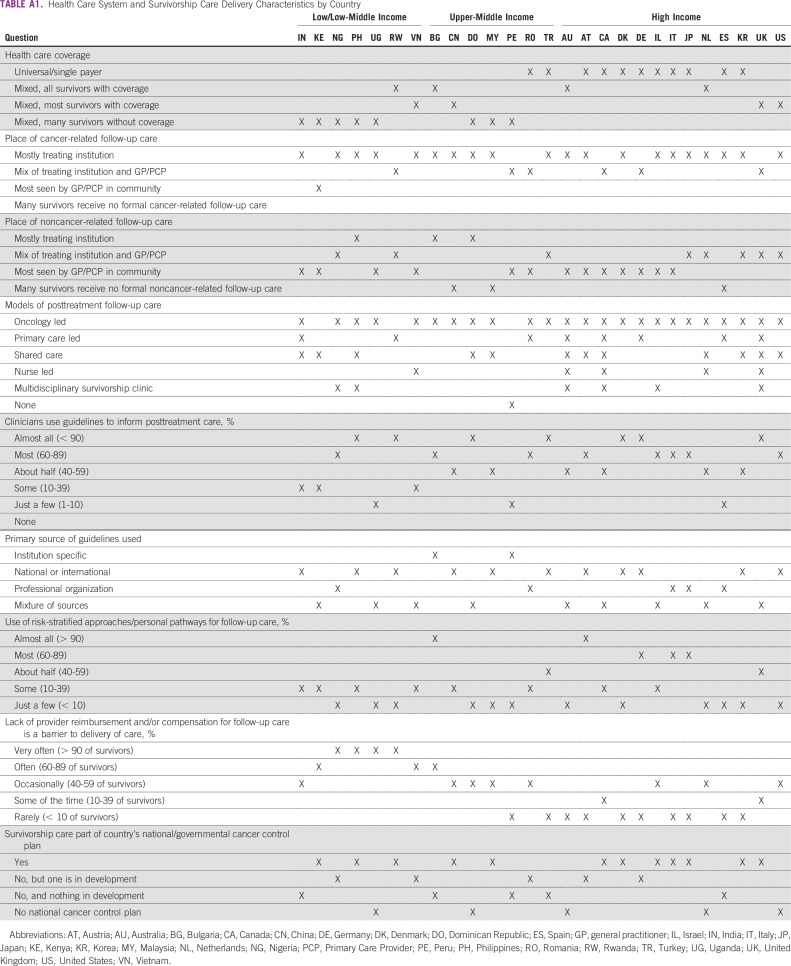
Health Care System and Survivorship Care Delivery Characteristics by Country

**TABLE A2:**
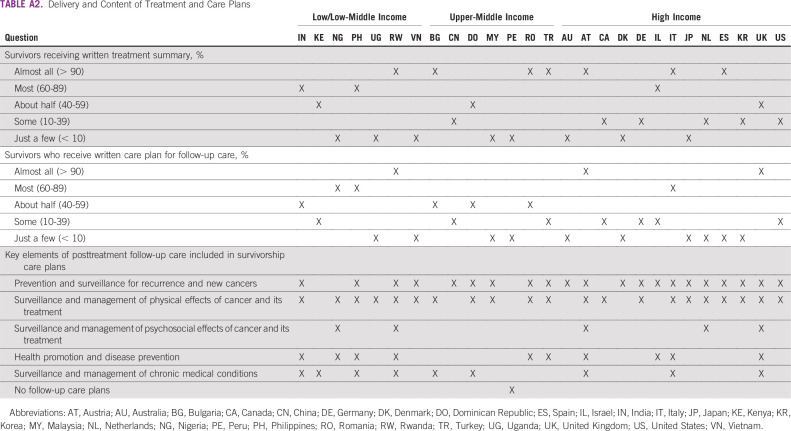
Delivery and Content of Treatment and Care Plans

**TABLE A3:**
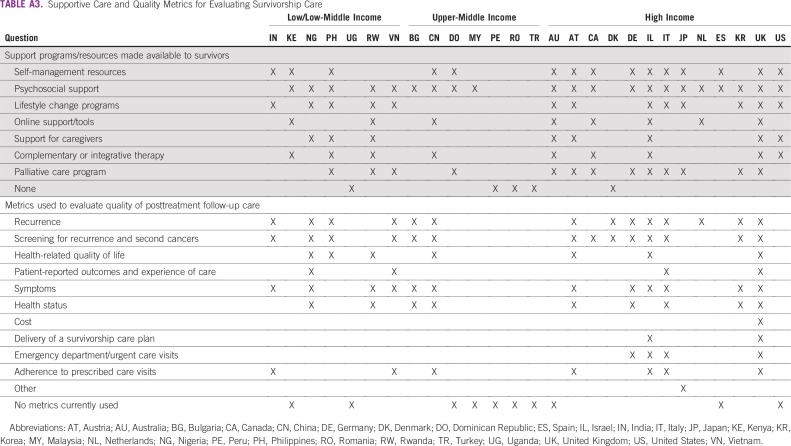
Supportive Care and Quality Metrics for Evaluating Survivorship Care

**TABLE A4:**
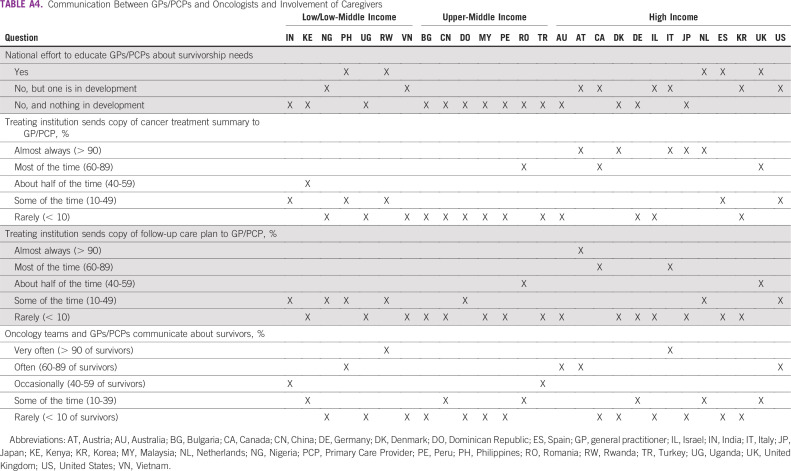
Communication Between GPs/PCPs and Oncologists and Involvement of Caregivers

## Appendix

### International Follow-Up Care for Breast and Colorectal Cancer Survivors Survey Questions

1. What type of health care coverage do cancer survivors have in your country? **(please select one)**
  ∘ Universal or single payer health care system that covers all cancer survivors
  ∘ Mixed payer system but all cancer survivors have some type of coverage
  ∘ Mixed payer system with most, but not all, cancer survivors having some type of coverage
  ∘ Mixed payer system with many cancer survivors not having coverage
2. What percentage of clinicians use guidelines to inform the posttreatment follow-up care of breast and colorectal cancer survivors? (**please select one**)
  ∘ Almost all (> 90%)
  ∘ Most (60%-89%)
  ∘ About half (40%-59%)
  ∘ Some (10%-39%)
  ∘ Just a few (1%-10%)
  ∘ None (please skip to question 4)
3. What is the primary source of guidelines used by clinicians for the care of breast and colorectal cancer survivors? (**please select one**)
  ∘ Institutional-specific set of guidelines
  ∘ National or international guidelines
  ∘ Professional organization guidelines (eg, ASCO)
  ∘ Mixture of guideline sources
  ∘ Other, please specify _____________________
4. Where are breast and colorectal cancer survivors seen for their routine cancer-related follow-up care visits? (please select one)
  ∘ ≥ 75% seen at the treating institution or a cancer center
  ∘ Between 25% and 75% seen at treating institution/cancer center and between 25% and 75% seen by a physician such as a general practitioner (GP) or primary care provider (PCP), in the community
  ∘ < 25% seen at the treating institution/ cancer center—most are seen by physician in the community, such as a GP or PCP
  ∘ < 25% seen at the treating institution/cancer center—many survivors receive no formal cancer-related follow-up care
5. Where are breast and colorectal cancer survivors seen for their routine noncancer-related follow-up care visits (including management of comorbid conditions)? **(please select one**)
  ∘ > 75% seen at the treating institution or a cancer center
  ∘ Between 25% and 75% seen at treating institution/cancer center and between 25% and 75% seen by a physician such as a GP or PCP, in the community
  ∘ < 25% seen at the treating institution/cancer center—most are seen by physician in the community, such as a GP or PCP
  ∘ < 25% seen at the treating institution/cancer center—many survivors receive no formal noncancer-related follow-up care
6. What percentage of institutions in your country use pathways or stratification based on patient needs and complexity when deciding which breast and colorectal cancer survivors are seen at the cancer center versus by physicians in the community? **(please select one)**
  ∘ Almost all (> 90%)
  ∘ Most (60%-89%)
  ∘ About half (40%-59%)
  ∘ Some (10%-39%)
  ∘ Just a few (< 10%)
7. What percentage of breast and colorectal cancer survivors in your country receive a **written summary of their cancer treatment**? **(please select one)**
  ∘ Almost all (> 90%)
  ∘ Most (60%-89%)
  ∘ About half (40%-59%)
  ∘ Some (10%-39%)
  ∘ Just a few (< 10%)
8. What percentage of breast and colorectal cancer survivors in your country receive a **written care plan** for posttreatment cancer-related follow-up care? **(please select one)**
  ∘ Almost all (> 90%)
  ∘ Most (60%-89%)
  ∘ About half (40%-59%)
  ∘ Some (10%-39%)
  ∘ Just a few (< 10%)
9. What percentage of survivorship programs involve nurses, nurse practitioners (NPs), and physician assistants (PAs) in providing follow-up care for breast and colorectal cancer survivors? **(please select one)**
  ∘ Almost all (> 90%)
  ∘ Most (60%-89%)
  ∘ About half (40%-59%)
  ∘ Some (10%-39%)
  ∘ Just a few (1%-10%)
  ∘ No nurses, NPs, or PAs involved in follow-up care for survivors
  ∘ No survivorship programs
10. Is there a national effort to educate GPs/PCPs about the needs and care of cancer survivors? **(please select one)**
  ∘ Yes
  ∘ No, but one is in development
  ∘ No, and nothing is in development
11. In general, how often does the treating institution send a copy of the **cancer treatment summary** to the GP/PCP? **(please select one)**
  ∘ Almost always (> 90%)
  ∘ Most of the time (60%-89%)
  ∘ About half of the time (40%-59%)
  ∘ Some of the time (10%-39%)
  ∘ Rarely (< 10%)
12. In general, how often does the treating institution send a copy of the **posttreatment cancer-related follow-up care plan** to the GP/PCP? **(please select one)**
  ∘ Almost always (> 90%)
  ∘ Most of the time (60%-89%)
  ∘ About half of the time (40%-59%)
  ∘ Some of the time (10%-39%)
  ∘ Rarely (< 10%)
13. In general, how often do oncology teams and GPs/PCPs communicate to share information about breast and colorectal cancer survivors? **(please select one)**
  ∘ Very often (> 90% of cancer survivors)
  ∘ Often (60%-89% of cancer survivors)
  ∘ Occasionally (40%-59% of cancer survivors)
  ∘ Some of the time (10%-39% of cancer survivors)
  ∘ Rarely (< 10% of cancer survivors)
14. What are the key elements of posttreatment follow-up care for breast and colorectal cancer survivors that oncology providers in your country routinely include in their follow-up care plans? (**select all that apply**)
  ∘ Prevention and surveillance for recurrence and new cancers
  ∘ Surveillance and management of physical effects of cancer and its treatment
  ∘ Surveillance and management of psychosocial effects of cancer and its treatment
  ∘ Health promotion and disease prevention
  ∘ Surveillance and management of chronic medical conditions
  ∘ Other: ________________________
  ∘ No follow-up care plans
15. What models of posttreatment follow-up care are used in your country? **(select all that apply)**
  ∘ Oncology-led follow-up care
  ∘ Primary care–led follow-up care
  ∘ Shared care (follow-up care is partnership between oncology team and primary care provider)
  ∘ Nurse-led follow-up care
  o Multidisciplinary survivorship clinic (care provided by a specialized team [ie, oncologist, psychologist, cardiologist, etc] in a separate clinical area)
  ∘ Other: _____________________________
  ∘ None
16. Are any of the following metrics used in your country to evaluate the quality of posttreatment follow-up care for breast and colorectal cancer? **(select all that apply)**
  ∘ Recurrence
  ∘ Screening for recurrence and second cancers
  ∘ Health-related quality of life
  ∘ Patient-reported outcomes and experiences of care
  ∘ Symptoms
  ∘ Health status
  ∘ Cost
  ∘ Delivery of a survivorship care plan
  ∘ Emergency Department/ urgent care visits
  ∘ Adherence to prescribed care visits/appointments
  ∘ No metrics currently used to evaluate the quality of posttreatment follow-up care
17. How often is lack of provider reimbursement/compensation for posttreatment follow-up care for breast and colorectal survivors a barrier to deliver these services? **(please select one)**
  ∘ Very often (> 90% of cancer survivors)
  ∘ Often (60%-89% of cancer survivors)
  ∘ Occasionally (40%-59% of cancer survivors)
  ∘ Some of the time (10%-39% of cancer survivors)
  ∘ Rarely (< 10% of cancer survivors)
18. What support programs/resources are made available to breast and colorectal cancer survivors after treatment ends? **(please select all that apply)**
  ∘ Self-management resources (for example: tools to provide survivors with information, advice and support to enable them to adapt to their condition)
  ∘ Psychosocial support (for example: counseling, support groups)
  ∘ Lifestyle change programs (for example: diet, exercise, smoking cessation)
  ∘ Online support/tools
  ∘ Support for caregivers
  ∘ Complementary or integrative therapy (for example: yoga, acupuncture)
  ∘ Palliative care program
  ∘ None
19. How often do oncology teams and GPs/PCPs communicate with family members as part of the patient’s follow-up care? (for example: addressing survivorship care needs)
  ∘ Almost always (>90%)
  ∘ Most of the time (60%-89%)
  ∘ About half of the time (40%-59%)
  ∘ Some of the time (10%-39%)
  ∘ Rarely (<10%)
20. Is care for cancer survivors part of your country’s National/Governmental Cancer Control Plan? **(please select one)**
  ∘ Yes
  ∘ No section on care for cancer survivors in National Cancer Control Plan, but one is in development
  ∘ No section on care for cancer survivors in National Cancer Control Plan, and nothing is in development
  ∘ No National Cancer Control Plan
21. Please tell us about the challenges in posttreatment follow-up care specific to country/survivor population.
